# Multiscale entropy and small-world network analysis in rs-fMRI — new tools to evaluate early basal ganglia dysfunction in diabetic peripheral neuropathy

**DOI:** 10.3389/fendo.2022.974254

**Published:** 2022-11-04

**Authors:** Geheng Yuan, Yijia Zheng, Ye Wang, Xin Qi, Rui Wang, Zhanyang Ma, Xiaohui Guo, Xiaoying Wang, Jue Zhang

**Affiliations:** ^1^ Department of Endocrinology, Peking University First Hospital, Beijing, China; ^2^ Academy for Advanced Interdisciplinary Studies, Peking University, Beijing, China; ^3^ State Key Laboratory of Media Convergence and Communication, Communication University of China, Beijing, China; ^4^ Neuroscience and Intelligent Media Institute, Communication University of China, Beijing, China; ^5^ Department of Plastic Surgery & Burns, Peking University First Hospital, Beijing, China; ^6^ Department of Radiology, Peking University First Hospital, Beijing, China; ^7^ College of Engineering, Peking University, Beijing, China

**Keywords:** basal ganglia, diabetic peripheral neuropathy (DPN), rs-fMRI (resting state fMRI), multiscale entropy (MSE), small-world network, early diagnostic marker, early dysfunction

## Abstract

**Objective:**

The risk of falling increases in diabetic peripheral neuropathy (DPN) patients. As a central part, Basal ganglia play an important role in motor and balance control, but whether its involvement in DPN is unclear.

**Methods:**

Ten patients with confirmed DPN, ten diabetes patients without DPN, and ten healthy age-matched controls(HC) were recruited to undergo magnetic resonance imaging(MRI) to assess brain structure and zone adaptability. Multiscale entropy and small-world network analysis were then used to assess the complexity of the hemodynamic response signal, reflecting the adaptability of the basal ganglia.

**Results:**

There was no significant difference in brain structure among the three groups, except the duration of diabetes in DPN patients was longer (p < 0.05). The complexity of basal ganglia was significantly decreased in the DPN group compared with the non-DPN and HC group (p < 0.05), which suggested their poor adaptability.

**Conclusion:**

In the sensorimotor loop, peripheral and early central nervous lesions exist simultaneously in DPN patients. Multiscale Entropy and Small-world Network Analysis could detect basal ganglia dysfunction prior to structural changes in MRI, potentially valuable tools for early non-invasive screening and follow-up.

## Introduction

Diabetic peripheral neuropathy (DPN) is the most common complications of diabetes mellitus, in relation to the degree and duration of diabetes, from 13% to 58% of patients with diabetes (1, 2). Previous studies have shown that patients with DPN are 15 times more likely to fall than healthy subjects, increasing the risk of fractures and other injuries, leading to high mortality in patients with DPN (3, 4). the central nervous system plays a critical role in balance control (5). Previous studies have mainly explored the issue of motor and balance impairments in DPN patients from the perspective of the behavioral and peripheral nervous system (PNS). However, the influence of the central nervous system(CNS) is unclear.

Although diabetic neuropathy has long been considered a disease of the PNS only, the involvement of the CNS in DPN is also gradually being realized (6–11). Recently, Magnetic Resonance Imaging (MRI) studies showed thalamus dysfunction in DPN patients (7–9, 12). Selvarajah et al. found that the volume of peripheral gray matter in DPN patients was significantly less than in patients with diabetes without DPN and in healthy participants; the decrease of gray matter was mainly concentrated in the primary somatosensory cortex, supramarginal gyrus, and cingulate cortex (6). These studies suggested that peripheral neuropathy was closely related to CNS disorders as a complete sensory motor control closed loop. Therefore, both CNS dysfunction and peripheral neuropathy may reduce balance control and increase the risk of falls in diabetic patients.

As a safe, non-invasive technique with high temporal and spatial resolution, functional magnetic resonance imaging(fMRI) is widely used to diagnose, predict and classify different stages of the disease (13, 14). Among these, resting-state functional magnetic resonance imaging (rs-fMRI) can reflect the degree of brain activity in the whole brain, making a breakthrough in exploring brain activity and brain functional connectivity (15–19). Particularly when gauging the progression of the disease, complex networks and complex systems analysis methods based on the resting blood oxygen level dependent (BOLD) signals are sensitive to subtle changes in functional connectivity and can be used to assess the early dysfunction of the cerebral cortex (20–26).

Basal ganglia, including globus pallidus, putamen, and caudate, is an essential hub in sensory and motor processing and plays a hub role in motor and balance performance (27–30). The putamen works in conjunction with the somatosensory cortex, external globus pallidus (GPe), internal globus pallidus (GPi), substantia nigra compacta (SNc) and substantia nigra reticulata (SNr) to control many types of motor skills. These include controlling motor learning, motor performance and tasks, motor preparation, specifying amplitudes of movement and movement sequences (30). Globus pallidus plays an important role in modulating involuntary movement, including standing, walking and speaking (29). Damage to the globus pallidus would result in movement disorder, and the modulation effects of the globus pallidus would also be weakened. In motor control, the globus pallidus could balance cerebellum excitability by reducing the excitability itself. Previous studies have observed basal ganglia lesions in diabetic uremic patients (31, 32). Moreover, we hypothesize that early CNS dysfunction (especially basal ganglia) appears in DPN patients. In the present study, multiscale entropy and small-world network analysis were performed to explore the complexity of the basal ganglia in DPN patients to assess the early CNS dysfunction.

## Materials and methods

### Participants

The diabetic patients in this study were all from the Department of Endocrinology, Peking University First Hospital. All patients were selected according to the following criteria (33): 1) type 2 diabetes, aged between 40 to 80 years, with no history of atherosclerotic cardiovascular disease (ASCVD) and stable glycemic control without therapy of DPN; 2) right-handed as determined by the Edinburgh Handedness Inventory; 3) ability to stand and walk normally determined by Berg Balance Scale(BBS) and Timed Up and Go test(TUG); 4) normal cognitive ability without antidepressant drugs determined by the Montreal Cognitive Assessment (MoCA); 5) no history of stroke, coronary disease, nephritis, tumors, gastrointestinal disease, psychiatric illness or peripheral neuropathy caused by excessive alcohol consumption, exposure to toxic substances and neurotoxicity; and 6) ability to meet the physical demands of the imaging procedure.

**Figure 1:**
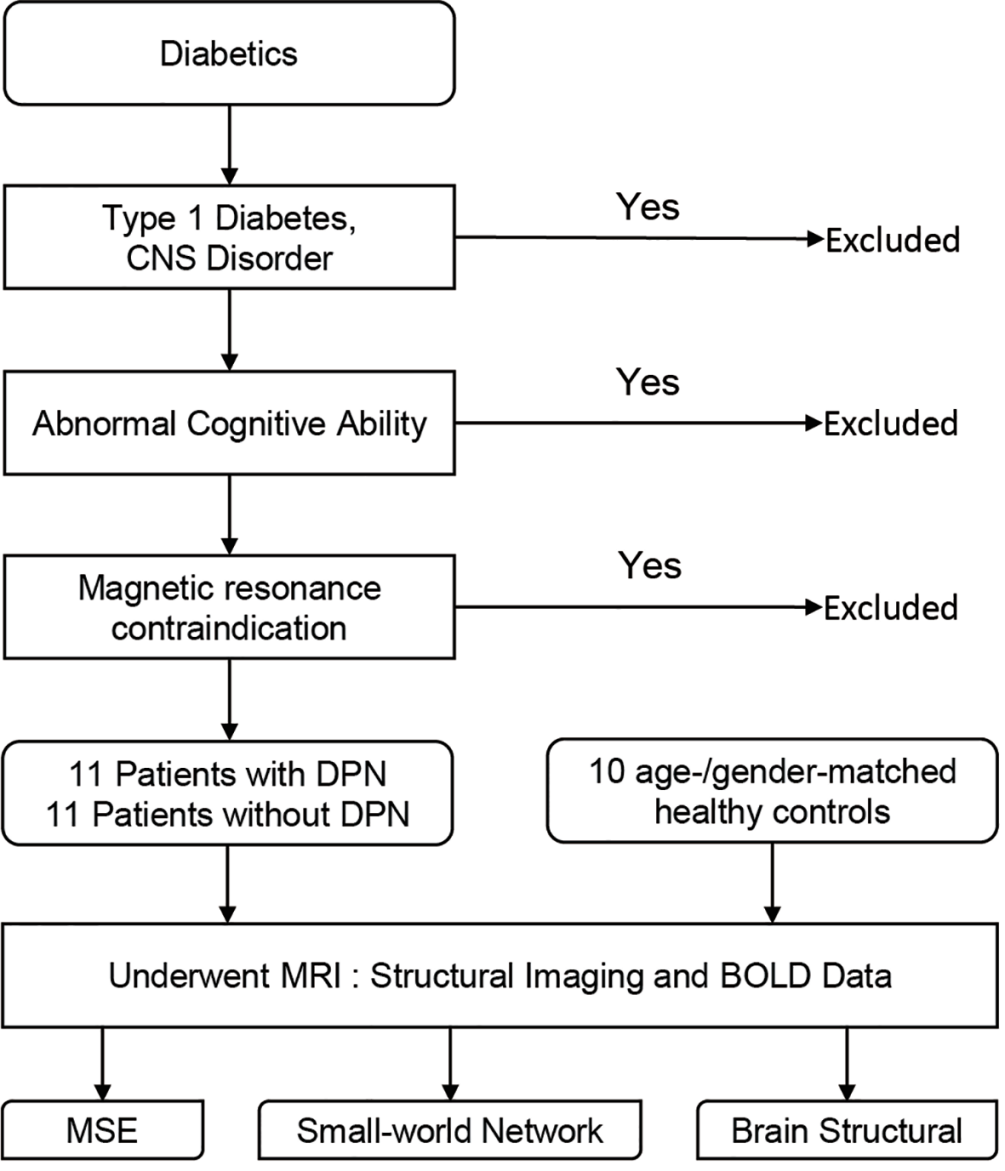
A flow chart of the study design.

According to the 2010 Toronto Diabetic Neuropathy Expert Group consensus panel (34), 11 patients with DPN (DPN group) confirmed by symptoms, physical examination, and nerve conduction velocity (NCV) test, and 11 diabetic patients without DPN (non-DPN group) were recruited. We also recruited 10 age- and gender-matched, right-handed, nondiabetic healthy controls (HC group). All control participants were free of CNS and PNS disorders. Then, all participants underwent MRI to assess the differences in brain structure, brain connectivity, and basal ganglia function. All participants were provided written informed consent for the protocol as approved by the Institutional Review Board of Peking University First Hospital, Beijing (2015[866]). A flow chart of this study design is shown in **Figure 1**.

### Cognitive ability test

All participants were subjected to a cognitive ability test that assessed their general mental status and other cognitive domains such as memory, attention, spatial processing, executive function, and language abilities. General mental status was assessed with the MoCA, and scores less than 26 were a reason for exclusion because participants with such low scores were considered to have possible dementia.

### Magnetic resonance image acquisition

The MRIs were acquired at the Peking University First Hospital using a GE 3T (Discovery MR750; GE Medical System) whole-body scanner with an eight-channel receive-only head coil. BOLD data were acquired using a standard echo-planar imaging sequence with the following parameters: repetition time/echo time, 2000/30ms; flip angle, 90°; image matrix, 64×64; thickness/spacing, 4mm/1mm; field of view, 230×230mm^2^; and 33 axial sections (35). We acquired 245 image volumes in each participant with their eyes open on a specific fixation point. High-resolution structural images were acquired by using a three-dimensional fast spoiled gradient echo sequence for anatomical localization (repetition time/echo time, 7.8/3.0ms; flip angles, 20°; inversion time, 450ms; field of view, 240×240mm^2^; slice thickness, 2mm with 1mm overlap; in-plane resolution, 1×1mm^2^).

### Data processing and analysis

One-way analysis of variances (ANOVAs) were used to assess the between-group differences (DPN, non-DPN, HC groups) in sex, age, height, weight, BMI, and MoCA score. A two-sample two-tailed t-test was carried out to test for significant differences in the HbA_1c_ and duration of diabetes between the DPN group and the non-DPN group. All statistical analyses were performed using SPSS version 24.0 for Windows software.

All brain structural images were processed to quantify and compare the difference in brain morphology between the DPN and non-DPN groups using the voxel-based morphometry (VBM) toolbox implemented in MATLAB (MathWorks, Inc., Sherborn, MA, U.S.A.), widely applied model in this field. First, the structure images were estimated and affine-transformed by VBM, and then a Gaussian kernel with FWHM (Full Width at Half Maximum) of 8 mm was used for spatial smoothing. Finally, A two-sample two-tailed t-test was conducted to test for significant structural differences between the two groups. The false discovery rate (FDR) approach was applied to correct multiple comparisons problem across space. P values <0.05, FDR corrected were considered significant.

Resting-state fMRI data were first preprocessed with Statistical Parametric Mapping software (SPM8, Wellcome Department of Imaging Neuroscience, University College London, UK) implemented in MATLAB. For each participant, images were realigned to the first scan to correct potential head movement within scans, generating six-parameter head motion curves. Each time-series was corrected to compensate for delays associated with acquisition time differences across slices. Functional images were co-registered to the corresponding structural T1 image and normalized to a 2-mm isovoxel Montreal Neurological Institute template. The first 5 data points were discarded because of the instability of initial MRI scanning, leaving 240 data points in the final data.

### Multiscale entropy and small-world network analysis

After preprocessing, Multiscale entropy (MSE) and small-world network analysis were used to assess the complexity of the hemodynamic response signal and network metrics of the region of interest (ROI) of the brain (related to sensorimotor processing, including PreCG: precentral gyrus; SMA: supplementary motor area; INS: insula; PoCG: postcentral gyrus; SPG: superior parietal gyrus; IPL: inferior parietal lobule; PCL: paracentral lobule; PUT: putamen; PAL: globus pallidum; THA: Thalamus).

MSE analysis is based on sample entropy (SampEn) that calculates SampEn on variable scales (36, 37). In this study, The registered fMRI data were segmented into 90 brain regions using the automated anatomical labeling (AAL) template. MSE of each AAL region were calculated with parameters of pattern length m = 2, distance threshold r = 0.3 and time scale τ = 4 (24, 38).

A small-world network is a network between the regular and random network (39). This study calculated network metrics using the GRETNA toolbox implemented in MATLAB. By traversing the connection threshold from 0.05 to 0.4 in increments of 0.01, the network parameters of the whole brain and local brain regions were calculated for each participant.

Finally, One-way ANOVAs were used to assess the between-group differences in MSE values, brain network parameters, and brain region connections. Tukey’s *post hoc* testing was used to analyze group differences within significant models.

## Results

### Baseline clinical characteristics

During the rs-fMRI testing, one participant in the DPN group and one in the non-DPN group were excluded because of involuntary excessive head motion. All information for 30 subjects (10 DPN, 10 non-DPN,10 HC) is shown in **Table 1**. The age in the DPN and non-DPN groups were 57.0 ± 9.8 and 56.6 ± 7.9 years(p<0.05) respectively. And the duration of DPN patients and non-DPN were 13.3 ± 6.8 and 7.8 ± 4.2 years (p<0.05),. The duration of diabetes in the DPN group was longer (p = 0.04). The HbA1c, MoCA score, age, and BMI were no differences among the three groups.

**Table 1:** Baseline information and characteristics of the all patients.

Characteristics	DPN (n=10)	Non-DPN (n=10)	HC (n=10)	p value
M/F	7/3	5/5	5/5	0.61
Age (years)	57.0 ± 9.8	56.6 ± 7.9	55.5 ± 5.5	0.91
Height (cm)	170.0 ± 6.2	169.9 ± 5.3	167.3 ± 6.4	0.53
Weight (kg)	72.6 ± 13.3	72.3 ± 10.0	62.0 ± 9.3	0.07
BMI	25.1 ± 4.7	25.1 ± 3.5	22.1 ± 2.9	0.15
MoCA score	27.8 ± 1.5	27.4 ± 1.4	27.9 ± 1.7	0.76
HbA_1c_ (%)	8.7 ± 1.7	8.9 ± 2.1	—	0.80
Duration of diabetes (years)	13.3 ± 6.8	7.8 ± 4.2	—	0.04

Values are given as mean ± SD, unless otherwise indicated.

### MSE analysis results

**Figure 2:**
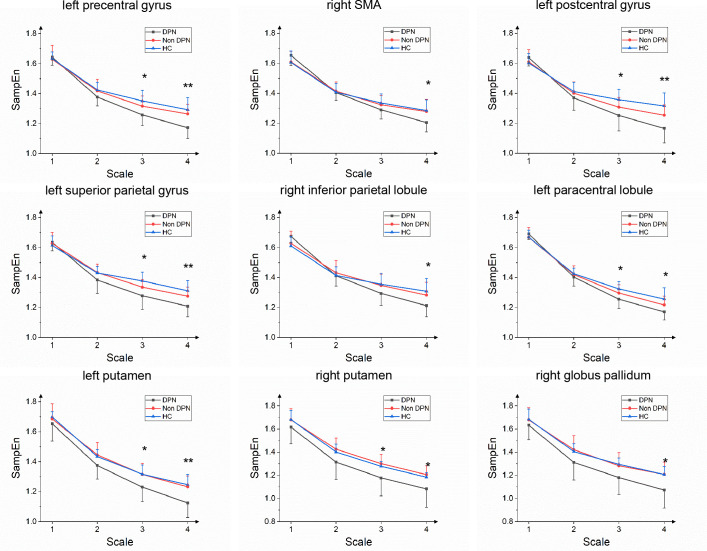
Group average SampEn over multiple time scales of left precentral gyrus, right supplementary motor area(SMA), left postcentral gyrus, left superior parietal gyrus, right inferior parietal lobule, left paracentral lobule, left and right putamen, right globus pallidum in the DPN group compared with the non-DPN group. (*, p<0.05; **, p<0.01).

The MSE values of each brain region were obtained on 4 scales, as shown in **Figure 2**. One-way ANOVAs showed that on a large scale, there were significant differences among groups in the left putamen, precentral gyrus, postcentral gyrus, superior parietal gyrus, paracentral lobule, right SMA, inferior parietal lobule, putamen, globus pallidum. Tukey’s *post hoc* testing showed that the MSE values of the DPN group in the left precentral gyrus, right supplementary motor area, left postcentral gyrus, left superior parietal gyrus, right inferior parietal lobule, left paracentral lobule, left putamen, and right globus pallidum was significantly lower than that of HC group (p<0.05). The MSE values of the DPN group in left and right putamen, left precentral gyrus and right globus pallidum were significantly lower than that of the non-DPN group (p<0.05). There was no significant difference between the non-DPN and HC groups in the brain regions mentioned above. No brain region in the DPN group showed a significant increase in complexity compared with the non-DPN group and HC group.

### Small-world network analysis results

**Figure 3:**
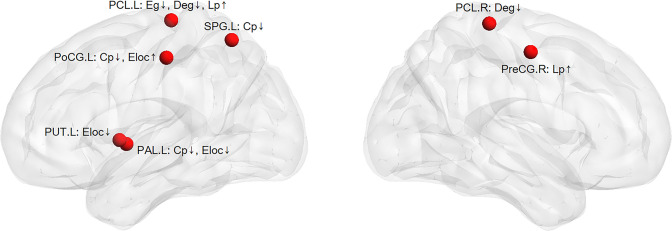
The weakened nodal network metrics in the DPN group compared with the non-DPN group and healthy control group. (PreCG, precentral gyrus; PoCG, postcentral gyrus; SPG, superior parietal gyrus; PCL, paracentral lobule; PUT, putamen; PAL, globus pallidum; Eloc, local efficiency; Cp, cluster coefficient; Eg, global efficiency; Lp, shortest path length; ↑ or ↓: the values of nodal network metrics in the DPN group are greater/smaller than those in the non-DPN group and the healthy control group).

**Figure 4:**
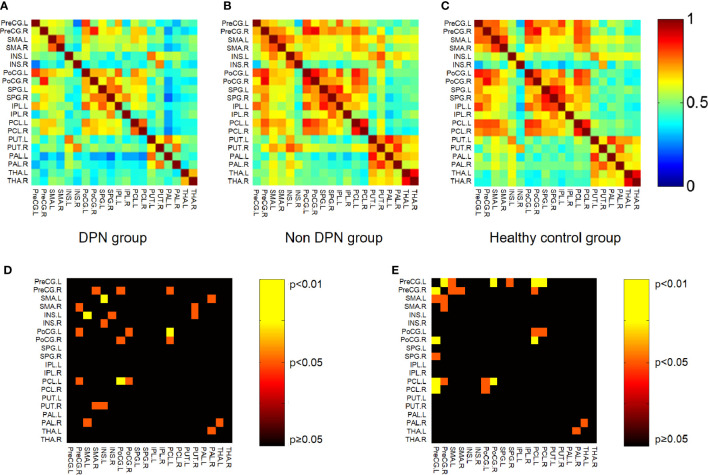
Connection matrix and weakened connections of brain region related to sensorimotor processing. **(A)** Brain connection matrix of the DPN group. **(B)** Brain connection matrix of the non-DPN group. **(C)** Weakened connections in regions related to sensorimotor processing in the DPN group compared with the non-DPN group. Warm color (non-DPN>DPN). **(D)** Weakened connections in regions related to sensorimotor processing in the DPN group compared with the non-DPN group. Warm color (non DPN>DPN). **(E)** Weakened connections in regions related to sensorimotor processing in the DPN group compared with the HC group. (PreCG, precentral gyrus; SMA, supplementary motor area; INS, insula; PoCG, postcentral gyrus; SPG, superior parietal gyrus; IPL, inferior parietal lobule; PCL, paracentral lobule; PUT, putamen; PAL, globus pallidum; THA, thalamus).

By calculating the brain network metrics, we found that, compared with the non-DPN group and healthy control group, the nodal importance in the bilateral paracentral lobule, left putamen, globus pallidum, superior parietal lobule, postcentral gyrus, right precentral gyrus was decreased (p<0.05), as shown in **Figure 3**. The brain connection matrix of the DPN group, non-DPN group, and HC group and the weakened connections of the DPN group are shown in **Figure 4**. Weakened connections between basal ganglia and sensorimotor cortex and between basal ganglia and thalamus were observed in the DPN group compared with the non-DPN and HC group (p<0.05).

### Brain structural analysis results

No significant changes in brain structure were found among the DPN group, non-DPN group, and HC group (FDR corrected, p>0.05).

## Discussion

This study explored the motor and balance impairments in patients with DPN from the perspective of CNS. The main finding of this study are as follows: (i) the basal ganglia adaptability was reducted without structural changes in DPN compared with in diabetic patients without DPN and healthy controls. (ii) Multiscale Entropy and Small-world Network Analysis could be valuable tools to evaluate early basal ganglia dysfunction.

As a nonlinear dynamics method, MSE analysis has several advantages. First, compared with SampEn, since some random fluctuations are removed, the entropy of random noise is lower in a large scale. Second, MSE reflects the scale irregularity of time series. If entropy decreases in scale, the sequence shows simple structures and randomness; if entropy increases in scale, the sequence shows high complexity. In 2013, Yang et al. used MSE for the first time to analyze resting-state BOLD signals (38). The results showed that MSE values were significantly lower in the left olfactory cortex, right posterior cingulate gyrus, right hippocampus, right parahippocampal gyrus, left superior occipital middle gyrus, left caudate nucleus, and left thalamus in older adults compared to younger adults. The human brain is a highly complex system that can be represented as a structurally or functionally interconnected network that assures rapid segregation and integration of information processing. Considerable progress has recently been made in describing the topological organization of the whole human brain’s networks using neuroimaging data and graph-theoretical approaches (20, 40). Liu et al. found that the small-world properties of the prefrontal, parietal and temporal lobes were significantly altered in schizophrenia patients compared to healthy subjects, and these changes correlate with illness duration in schizophrenia (41).

Multiscale entropy (MSE) and small-world network analysis were used to assess the complexity of hemodynamic response signal and network metrics of brain regions among diabetic patients with and without DPN and healthy elderly. The results showed that the DPN group significantly reduces the complexity of the left putamen, right putamen, and right globus pallidum. Complexity reflects the system’s adaptability, so the complexity reduction of the left putamen, right putamen, and right globus pallidum is probably closely related to their reduced adaptability. Moreover, the local network efficiency of the left putamen and globus pallidum and the cluster coefficient of the left globus pallidum showed remarkably reduced, indicating that their information transmission capacity decreased and connection with neighboring nodes weakened in the DPN group. Also, this study suggested that the connection between the thalamus and globus pallidum was weakened in the DPN group. It is well known that the decreased complexity and reduced network robustness are closely associated with declined adaptability of the cerebral cortex (20, 36, 40, 42, 43). Therefore, basal ganglia adaptability was reduced in patients with diabetic peripheral neuropathy. There was no significant difference in brain structure among the three groups in our study. Because the age and duration of diabetes in our study were relatively shorter than in previous studies, we speculate that the functional changes appear earlier than structural changes. This finding is consistent with recent findings that AD is not only associated with the gray and white matter atrophy but also with changes in the connectivity of brain regions prior to any structural changes (14, 26). Thus basal ganglia dysfunction could be used as an early diagnostic marker, which is essential for early screening in DPN patients.

The main finding of this study is reduced basal ganglia adaptability in DPN patients, but the underlying etiology for reduced adaptability of the basal ganglia is not clear. In this study, DPN patients, age-matched non-DPN patients, and healthy controls were recruited to eliminate the effects of age. Therefore, peripheral neuropathy and the increasing duration of diabetes might be the primary etiology of the reduced adaptability of the basal ganglia in DPN patients. These findings and recent studies demonstrate that peripheral and central nervous lesions exist simultaneously in DPN patients. However, the temporal involvement relationship between PNS and CNS is challenging to elucidate. One possibility is that loss of the afferent input, resulting from peripheral nerve damage, subsequently causes changes at progressively higher levels in the CNS, especially in the basal ganglia, the important somatosensory and motor processing hub. The other is also possible that the long-term metabolic abnormalities result in reduced adaptability of the basal ganglia. The basal ganglia changes could be occurring with changes in the PNS previously or concomitantly. The coupling of PNS and CNS builds up the sensorimotor loop, and both possibilities suggest the sensorimotor ‘dying-back’ mechanism in DPN. Further longitudinal studies will be needed to explore the temporal relationship between PNS and CNS involvement in DPN.

Resting-state functional magnetic resonance imaging (rs-fMRI) can reflect the degree of brain neuron activity and has been widely used in the field of the central nervous system. Previous studies have focused on brain morphology and central responses to specific stimuli based on rs-fMRI in DPN patients (17, 18, 44). As mentioned above, MSE and small-world network analysis based on resting BOLD signals are sensitive to assessing the cerebral cortex’s adaptability by analyzing the complexity of the resting BOLD signal, brain network characteristics, and brain function connections (20–26). For example, small-world network analysis has reported AD-induced changes in global brain functional connectivity in early Alzheimer’s disease(AD) (26). Thus, the brain dysfunction of DPN patients from the complex network and complex system perspectives should be explored. This study showed that the basal ganglia adaptability was reduced in patients with diabetic peripheral neuropathy by MSE and small-world network analysis based on the rs-fMRI. These tools are potentially a non-invasive marker for early diagnosis and long-term follow-up in diabetics by assessing the early basal ganglia dysfunction.

Further studies could provide early screening for basal ganglia adaptability in patients with long-standing diabetes and thus indicate whether they need targeted diagnostic and treatment strategies. Deep-Brain Stimulation (DBS) has become an accepted treatment for basal ganglia disorders (45, 46). Therefore, we think that brain stimulations, including DBS, transcranial magnetic stimulation (TMS), and transcranial direct current stimulation(tDCS), targeting basal ganglia, may help to improve the motor balance control by enhancing the adaptability of basal ganglia in patients with diabetes, especially for DPN patients. Except for tight glucose control, physical therapy, such as transcutaneous electrical nerve stimulation (TENS), gait training, and exercise programs can reduce pain, maintain strength, and improve motor and balance function in DPN patients (47, 48). Moreover, recent studies showed that liraglutide, as a small molecule, could directly affect neurons in the brain to prevent both motor dysfunction in the substantia nigra and basal ganglia (49, 50). Therefore, it might be helpful in future research and clinical treatment to improve motor and balance control and reverse the changes of diabetic neuropathy in DPN patients by combining physical therapy, drugs and brain stimulations targeting basal ganglia.

## Conclusion

In this study, we demonstrate that the adaptability of basal ganglia is reduced, and the stability of the sensorimotor loop is decreased in DPN patients compared with diabetic patients without DPN. These findings demonstrate that peripheral and central nervous lesions co-exist as an early CNS disorder in DPN patients. Prior to structural changes, the basal ganglia dysfunction in DPN patients could be evaluated early by multiscale entropy and small-world network analysis. Thus, the study has the potential to provide a non-invasive practical tool for early diagnosis and long-term follow-up in diabetics by assessing the early basal ganglia dysfunction.

## Data availability statement

The datasets presented in this article are not readily available because due to privacy and ethical concerns, neither the data nor the source of the data can be made available. Requests to access the datasets should be directed to Jue Zhang, zhangjue@pku.edu.cn.

## Ethics statement

The studies involving human participants were reviewed and approved by the Institutional Review Board of Peking University First Hospital, Beijing (2015[866]). The patients/participants provided their written informed consent to participate in this study.

## Author contributions

GY, YW, and XQ designed the study. YZ, YW, RW, and ZM analyzed the data. GY, YZ, and YW wrote the manuscript. XG, XW, and JZ overviewed the paper. All authors contributed to the article and approved the submitted version.

## Funding

This work was supported by grants from the National Natural Science Foundation of China (Grant Nos. 11372013 and 11572003).

## Acknowledgments

The authors thank all the participants for participating in the study.

## Conflict of interest

The authors declare that the research was conducted in the absence of any commercial or financial relationships that could be construed as a potential conflict of interest.

The reviewers QP and LX declared a shared affiliation with the authors to the handling editor at the time of review.

## Publisher’s note

All claims expressed in this article are solely those of the authors and do not necessarily represent those of their affiliated organizations, or those of the publisher, the editors and the reviewers. Any product that may be evaluated in this article, or claim that may be made by its manufacturer, is not guaranteed or endorsed by the publisher.
